# NRF2 Activation Ameliorates Oxidative Stress and Improves Mitochondrial Function and Synaptic Plasticity, and in A53T α-Synuclein Hippocampal Neurons

**DOI:** 10.3390/antiox11010026

**Published:** 2021-12-24

**Authors:** Mikah S. Brandes, Jonathan A. Zweig, Anita Tang, Nora E. Gray

**Affiliations:** Department of Neurology, Oregon Health & Science University, Portland, OR 97239, USA; brandes@ohsu.edu (M.S.B.); zweigj@ohsu.edu (J.A.Z.); anitatang99@gmail.com (A.T.)

**Keywords:** alpha-synuclein, NRF2, dimethyl fumarate, Parkinson’s disease

## Abstract

In Parkinson’s disease (PD), brain oxidative stress and mitochondrial dysfunction contribute to neuronal loss as well as motor and cognitive deficits. The transcription factor NRF2 has emerged as a promising therapeutic target in PD because it sits at the intersection of antioxidant and mitochondrial pathways. Here, we investigate the effects of modulating NRF2 activity in neurons isolated from a A53T α-synuclein (A53TSyn) mouse model of synucleinopathy. Embryonic hippocampal neurons were isolated from A53TSyn mice and their wild type (WT) littermates. Neurons were treated with either the NRF2 activator dimethyl fumarate (DMF) or the NRF2 inhibitor ML385. Reactive oxygen species (ROS), dendritic arborization and dendritic spine density were quantified. Mitochondrial bioenergetics were also profiled in these neurons. A53TSyn neurons had increased ROS and reduced basal and maximal mitochondrial respiration relative to WT neurons. A53TSyn neurons also displayed decreased dendritic arborization and reduced spine density. Treatment with DMF reduced ROS levels and improved both mitochondrial function and arborization, while inhibition of NRF2 with ML385 exacerbated these endpoints. Modulation of NRF2 activity had a significant effect on mitochondrial function, oxidative stress, and synaptic plasticity in A53TSyn neurons. These data suggest that NRF2 may be a viable target for therapeutic interventions in PD.

## 1. Introduction

While motor symptoms are the predominant clinical manifestation of Parkinson’s Disease (PD), cognitive impairment also occurs in the majority of cases as the disease progresses, affecting as many as 80% of patients who have had the disease 15 years or longer [1]. Yet, cognitive impairment is often overlooked as a target for therapeutic intervention. Increased oxidative stress [2], diminished mitochondrial function [3] and neuronal loss contribute to both motor and cognitive symptoms in PD [4,5,6]. The accumulation of aggregated α-synuclein (aSyn) found in Lewy bodies is the pathological hallmark of PD and believed to contribute to the degeneration of neurons [5]. Aggregated aSyn has also been shown to increase reactive oxygen species (ROS) and disrupt mitochondrial function [7].

Multiple studies have demonstrated a relationship between mitochondrial function, antioxidant capacity and cognitive function. In mice, cognitive decline is associated with dysfunctional mitochondria [8], increased oxidative damage [9], and decreased brain and plasma antioxidants [10,11,12], whereas over-expressing mitochondrial antioxidant enzymes has been shown to improve memory in rodents [13,14,15]. These observations have further underscored the therapeutic potential of targeting both antioxidant and mitochondrial pathways to improve cognitive function.

The transcription factor NRF2 (nuclear factor erythroid 2-related factor 2, also called NFE2L2) regulates the endogenous antioxidant response pathway by binding to antioxidant response elements (AREs) in the promoters of target genes and modulating the expression of antioxidant enzymes [16]. NRF2 has also been shown to regulate the expression of mitochondrial proteins as well [17]. The increased mitochondrial dysfunction and oxidative stress seen in the PD brain suggest that NRF2 may be a promising target for therapeutic intervention. This idea is supported by findings from a genome-wide association study that found that a functional haplotype in the human NFE2L2 promoter that results in increased transcriptional activity of the NRF2 was associated with decreased risk and delayed onset of PD [18].

Further evidence supporting a role for targeting NRF2 in PD is the fact that loss of NRF2 in an aSyn overexpressing mouse was shown to result in increased aSyn accumulation and neuronal death [19,20]. In our own lab we have found aged mice that do not express NRF2 have impaired hippocampal mitochondrial function, decreased expression of synaptic proteins and impaired cognitive function relative to wild-type (WT) animals [21]. We saw similar effects in neurons isolated from those mice including impaired mitochondrial function as well as decreased synaptic density and reduced dendritic arborization [22]. Conversely, activation of NRF2 by a variety of compounds has been shown to induce antioxidant response, improve mitochondrial health, enhance synaptic density and be neuroprotective in in vitro models of other neurodegenerative diseases [23]. These same effects are seen with in vivo NRF2 activation along with enhanced cognitive function [23].

In this study we take the first step towards evaluating the therapeutic potential of targeting NRF2 for cognitive enhancement in PD by exploring effects of modulating NRF2 activity on the physiological underpinnings of improved cognitive function, namely synaptic density. In humans, decreased synaptic density is associated with diminished cognitive capacity [24] and we have seen in our own lab that dendritic spine loss is also correlated with reduced cognitive performance in mice [25]. Here, in addition to evaluating the effects of NRF2 activity on dendritic arborization and spine density we will also investigate mitochondrial and antioxidant effects in hippocampal neurons isolated from the A53TSyn mouse model of synucleinopathy.

## 2. Materials and Methods

### 2.1. Culture of Primary Hippocampal Neurons

Mice were housed in an AALAC certified facility and maintained in a climate-controlled environment with a 12-h light/12-h dark cycle and fed a Pico Lab Rodent Diet 5LOD (LabDiets, St. Louis, MO, USA). Diet and water were supplied ad libitum. All procedures were conducted in accordance with the NIH Guidelines for the Care and Use of Laboratory Animals and were approved by the institutional Animal Care and Use Committee of Oregon Health and Science University.

Hippocampal neurons were isolated from A53TSyn embryonic mice, based on the methods of Kaech and Banker [26]. eGFP-A53TSyn transgenic mice were generously donated by Dr Vivek Unni [27]. The Unni lab has shown that the GFP tag in no way interferes with the aSyn protein dynamics or accumulation [28,29]. These mice were bred to C57BL6 mice acquired from Jackson Laboratories. Embryos were harvested at 18 days of gestation from anesthetized females. A53T positive progeny were phenotypically distinguished by be exposure to a fluorescent light source. Hippocampi were dissected, gently minced, trypsinized, and triturated to generate suspensions of dispersed neurons.

### 2.2. Analysis of Dendritic Arborization

Sholl analysis was used to assess dendritic complexity. Neurons were plated at a density of 130,000 in 60 mm dishes in MEM medium (GIBCO/Life Technologies, Waltham, MA, USA), 5% FBS (Atlanta Biologicals, Flowery Branch, GA, USA), 1× Anti-Anti (GIBCO/Life Technologies) and 0.6% glucose (Sigma-Aldrich, St. Louis, MO, USA), each dish containing 3 poly-l-lysine-coated nitric acid treated glass coverslips with paraffin wax spacers. After 3 h, the coverslips were flipped into 60 mm dishes containing mouse neural stem cell-derived glial cells (provided by Dr. Gary Banker, Jungers Center, OHSU) and maintained in 6 mL Neurobasal Medium supplemented with 1× GlutaMAX (GIBCO/Life Technologies), 1× Anti-Anti (GIBCO/Life Technologies) and 1× GS21 neural supplement (ThermoFisher, Waltham, MA, USA). Dishes were fed every week by removing 1 mL of the culture medium and adding 1 mL fresh Neurobasal medium that included GlutaMAX, Anti-Anti and Neuronal Culture Medium Supplement, with the first feed at 5 days in vitro (DIV) containing 6 μM (1 μM Final) cytosine β-d-arabinofuranoside hydrochloride (AraC; Sigma-Aldrich). Coverslips were fixed in 4% PFA in PBS at 12, 19, or 26 DIV and treated with DMSO as a vehicle control, 20 μM DMF or 1 μM ML385 for 7 days prior to fixation. Coverslips were stained with Anti-MAP2B (Sigma-Aldrich #M4403; 3.3 μg/mL) and Goat anti-mouse IgG1-Cy3 (Jackson ImmunoResearch #115-165-205; 1.5 μg/mL). Immunostained neurons were imaged with a Zeiss ApoTome2 microscope and blinded Sholl analyses were performed using the Fiji platform. Thirty isolated, non-overlapping cells were analyzed per coverslip. Arborization data was pooled across 3 independent experiments (3–5 coverslips per genotype and treatment condition in each experiment) providing at least 300 cells per genotype.

### 2.3. Analysis of Dendritic Spine Density

For the analysis of dendritic spine density, 150,000 hippocampal neurons were electroporated with plasmids encoding enhanced Green Fluorescent Protein (eGFP) and plated onto dishes with coverslips containing 300,000 WT or A53TSyn cortical neurons per dish. Cortical neurons were plated 7 days prior to the addition of the hippocampal neurons. This strategy promoted robust FIJI software.

### 2.4. Cell Viability Determination

Cell viability was determined using the CellTiter 96 Aqueous Non-Radioactive Cell Proliferation Assay (Promega, Madison, WI, USA) as per the manufacturer’s instructions. Neurons were plated at a density of 25,000 cells per well of a poly-l-lysine coated 96-well plate and grown for 5 days in Neurobasal media with GS21, Anti-Anti and GlutaMAX. Cells were then treated with increasing concentrations of DMF or ML385 and viability was quantified two days later. Assays were conducted with 4–8 wells per treatment condition per plate. The assays were repeated 3–4 times yielding a total of 16–24 replicates per treatment condition.

### 2.5. NRF2 Activation Assay in HepG2-ARE Reporter Cells

HepG2 cells that stably express a firefly luciferase gene under the control of the ARE promoter were obtained from BPS Bioscience. Cells were grown in MEM medium supplemented with 10% FBS, 1% non-essential amino acids, (Life Technologies, Waltham, MA, USA), 1 mM sodium pyruvate and 1% penicillin/streptomycin. Cells were plated at a density of 30,000 per well in a 96-well plate and treated for 48 h with increasing concentrations of either DMF or ML385 and NRF2 activity was quantified using the Pierce Firefly Luc One-Step Glow Assay Kit (Thermo) as per the manufacturer’s instructions. Luminescence was normalized to total protein content as determined by a bicinchoninic acid (BCA) assay.

### 2.6. Analysis of Mitochondrial Function

Mitochondrial function was evaluated using the Seahorse Bioscience XFe96 Extracellular Flux Analyzer. aSyn neurons were plated at a density of 60,000 cells/well on 96 well Seahorse culture plates (Agilent Technologies, Santa Clara, CA, USA) in DMEM/F12 containing N2 growth supplement. After 5 days, cells were treated with either DMSO, DMF (20 µM) or ML385 (1 µM). Two days later cells were switched into assay medium (pH 7.4) containing XF Base medium (Seahorse Bioscience), 5.5 mM glucose and 1mM sodium-pyruvate and analyzed using the MitoStress Kit as previously described [30]. Neurons can survive roughly 10–14 days without a feeder layer of glial cells, so taking measurements after 7 days allowed us to capture changes that occur in the cell before viability becomes an issue. Oxygen consumption rate (OCR) was measured under varying conditions. After three initial baseline measurements of OCR, the ATP synthase inhibitor oligomycin (1 μM) was added and three subsequent measurements were taken. Next an ETC accelerator, p-trifluoromethoxy carbonyl cyanide phenyl hydrazone (FCCP at 1.5 μM), was added and after 3 measurements were taken, mitochondrial inhibitors rotenone (1 μM) and antimycin (1 μM) were added, and three final measurements were taken. Data was normalized to total DNA content, which was determined from each well using the CyQuant kit (Invitrogen, Waltham, MA, USA) as per the manufacturer’s instructions.

### 2.7. ROS Quantification

A53TSyn hippocampal neurons were plated at a density of 75,000 cells per well in a lysine coated 96-well plate and allowed to grow in Neurobasal medium with GlutaMAX and GS21 for 5 days, followed by treatment with either DMSO, DMF (20 µM) or ML385 (1 µM) for 2 days. ROS content was assessed by a Cellular ROS Assay Kit (Abcam 113851) as per the kit’s instructions. A BCA was used to normalize the values to the total protein content of each well. Data was collected across three independent experiments with at least 6 wells per genotype in each experiment.

### 2.8. Gene Expression

Neurons were plated at a density of 250,000 cells per well in lysine coated 12-well plates. RNA was extracted using Tri-Reagent (Molecular Research Center, Cincinnati, OH, USA) and reverse transcribed with the Superscript III First Strand Synthesis kit (Invitrogen) to generate cDNA.

Gene expression was determined using TaqMan Gene Expression Master Mix (Invitrogen) and commercially available TaqMan primers (Invitrogen) for Kelch-like ECH-associated protein 1 (*Keap1*), NAD(P)H Quinone Dehydrogenase 1 (*Nqo1*), Heme oxygenase 1 (*Hmox1*), Glutamate-Cysteine Ligase Catalytic Subunit (*Gclc*) and glyceraldehyde 3-phosphate dehydrogenase (*Gapdh*). Quantitative PCR (qPCR) was carried out on a StepOne Plus Machine (Applied Biosystems) and gene expression was analyzed using the delta-delta Ct method normalizing to expression of *Gapdh*.

### 2.9. Statistics

Statistical significance was calculated using student’s *t*-tests for two-way comparisons or ANOVA followed by pairwise post hoc testing, for comparisons of more than two groups. Significance was defined as *p* ≤ 0.05. Analyses were performed using Excel or GraphPad Prism8.

## 3. Results

### 3.1. Determination of Non-Lethal Concentrations of DMF and ML385

**Figure 1:**
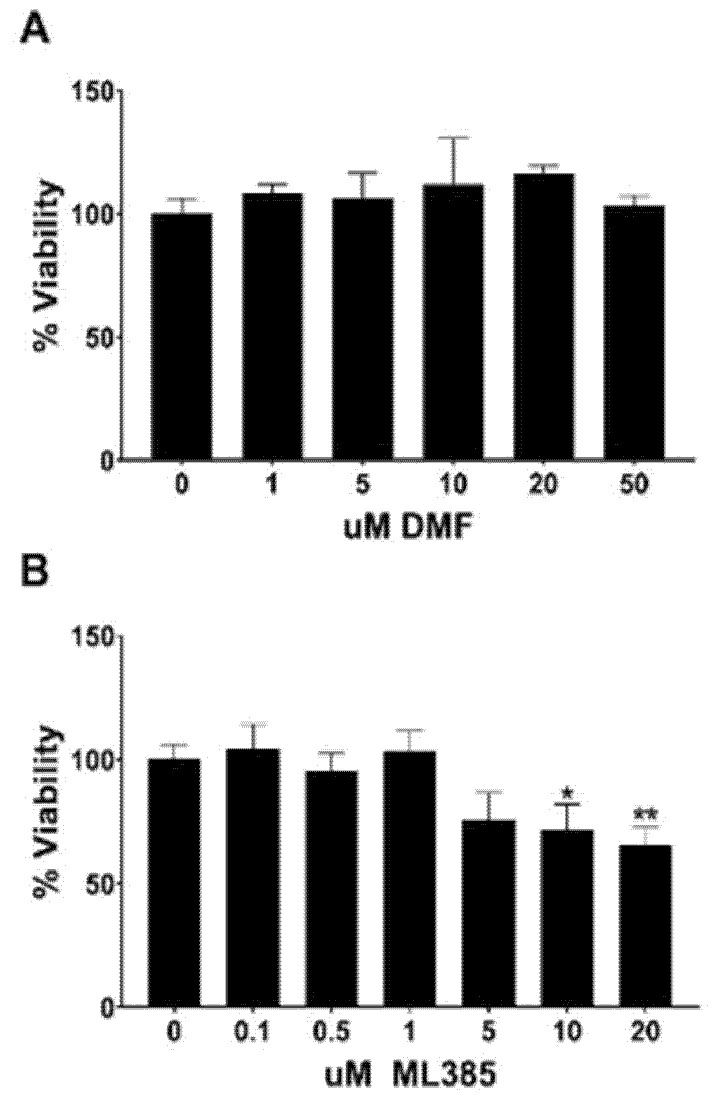
Cytotoxicity of DMF and ML385. (**A**) No cell death was observed at any of the concentrations of DMF tested whereas (**B**) significant cytotoxicity was seen when cells were treated with concentrations of ML385 of 10 µM or higher. Significance is relative to control (* p < 0.05, ** *p* < 0.01).

To determine the appropriate concentration of DMF or ML385 to be used in our experiments, WT hippocampal neurons were treated for 48 h with the NRF2 activating compound, DMF, at concentrations ranging from 1 to 50 µM. No significant cell death was observed at any concentration (Figure 1A). WT neurons were also treated with the NRF2 inhibitor ML385 at concentrations ranging from 0.1 to 20 µM. Significant toxicity was evident at 10 and 20 µM ML385 (Figure 1B).

### 3.2. NRF2 Activation by DMF and Inhibition by ML385 and NRF2-Regulated ARE Gene Expression

**Figure 2:**
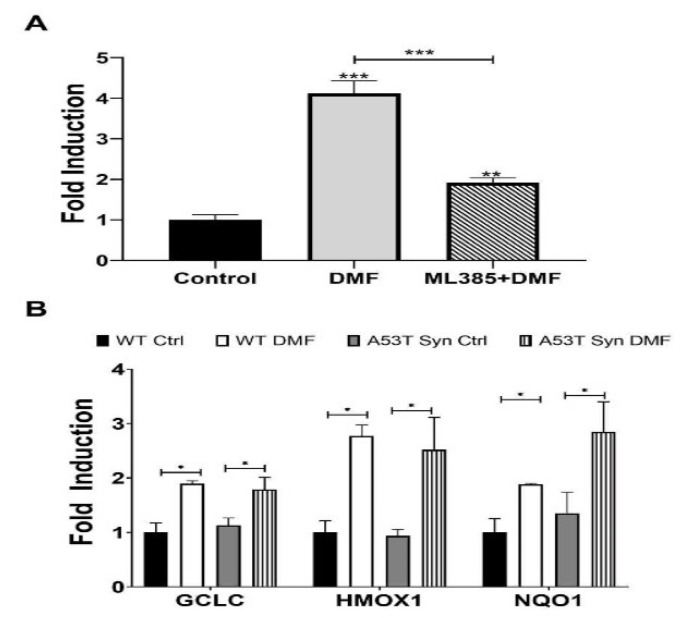
DMF activates NRF2 in vitro and this activation is inhibited by ML385. (**A**) 20 µM DMF significantly induces NRF2 expression in HepG2-ARE cells, and co-treatment with 1 µM ML385 significantly inhibits this effect. (**B**) 20 µM DMF significantly induced expression of NRF2-ARE genes, GCLC, HMOX1, and NQO1 in WT and A53TSyn neurons (*n* = 10–12). Significance is relative to control unless otherwise indicated (* p < 0.05, ** *p* < 0.01, *** *p* < 0.001).

The HepG2-ARE cell line express a firefly luciferase gene under the control of the ARE promoter and therefore can be used to assess NRF2 activation following compound treatment. Robust NRF2 activation was seen following 48 h of treatment with 20 µM DMF. Co-treatment with 1 µM ML385 attenuated this activation (Figure 2A). A similar activation following DMF treatment was observed in primary hippocampal neurons treated with DMF. Expression of the NRF2 target *Gclc*, *Hmox1*, and *Nqo1* was increased by DMF treatment in both WT and A53TSyn (Figure 2B).

Because NRF2 activity is heavily regulated through binding with KEAP1, we also evaluated *Keap1* gene expression in order to determine if there were differences in basal expression between WT and A53TSyn neurons. We found that there was no difference in expression between the two genotypes (Supplementary Figure S1).

### 3.3. NRF2 Activity Alters Mitochondrial Function and Oxidative Stress in A53TSyn Neurons

**Figure 3:**
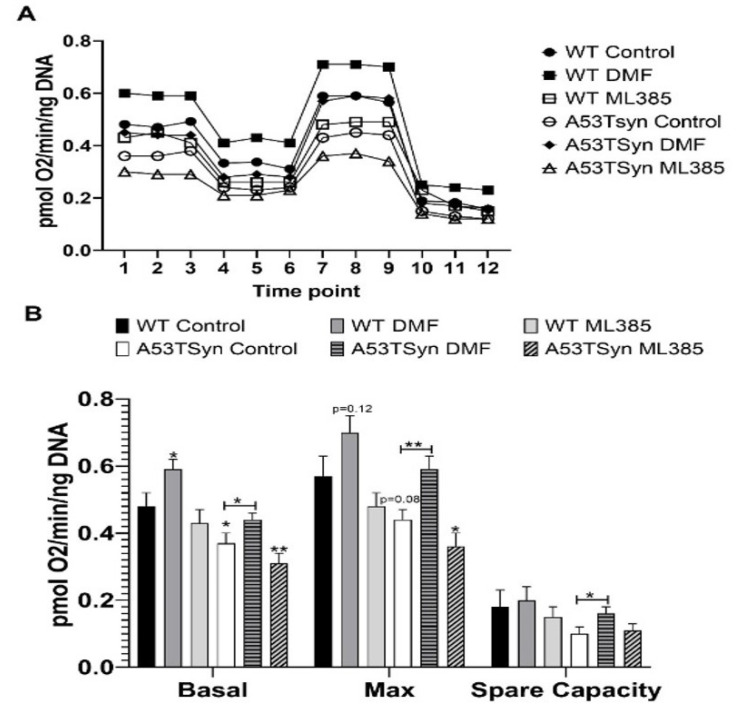
Deficits in mitochondrial respiration in A53TSyn neurons are attenuated by DMF and exacerbated by ML385. (**A**) Differences were observed in the bioenergetic profile of A53TSyn and WT neurons and further altered by treatment with DMF and ML385. (**B**) DMF treatment increased basal and maximal respiration as well as spare capacity in A53TSyn neurons. In WT neurons basal respiration was increased by DMF but otherwise unaffected by treatment. Significance is relative to control unless otherwise indicated (* p < 0.05, ** *p* < 0.01).

A53TSyn hippocampal neurons showed a deficit in mitochondrial bioenergetic profile relative to WT neurons. These deficits were attenuated by DMF treatment or exacerbated by ML385 administration (Figure 3A). Basal respiration, the average of the three initial readings, in A53TSyn neurons was significantly lower than that of WT neurons (Figure 3B). DMF treatment attenuated this deficit in A53Tsyn neurons and increased basal oxygen consumption rates in WT neurons as well. ML385 treatment also reduced basal respiration in A53TSyn neurons but had no effect on basal respiration in WT neurons (Figure 3B). A similar, though non-significant, trend toward reduced oxygen consumption in A53Tsyn neurons relative to WT was also seen in maximal respiration, the average of the three readings following FCCP addition, as well as spare capacity. DMF treatment significantly increased maximal respiration in A53TSyn neurons and although not statistically significant, resulted in a trend towards increased maximal respiration in WT neurons. The difference between the maximal oxygen consumption rate and the basal oxygen consumption rate is the spare capacity of the cell and reflects the amount of extra ATP that can be generated in response to a sudden increase in energy demand. DMF treatment increased spare capacity in A53TSyn neurons but ML385 had no effect on spare capacity. Neither treatment affected spare capacity in WT neurons.

**Figure 4:**
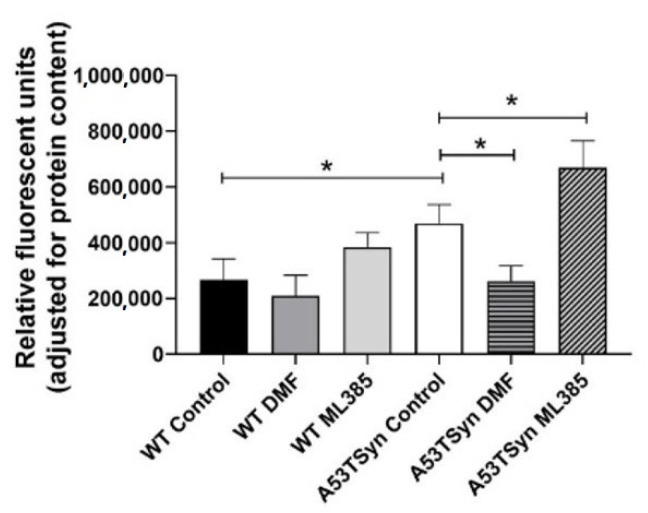
DMF attenuates elevated intracellular ROS in A53TSyn neurons. Levels of intracellular ROS were elevated in A53TSyn neurons relative to WT, and this increase was attenuated by treatment with DMF (20 µM). ML385 (1 µM) exacerbated the increased ROS in A53TSyn neurons. Significance is relative to control unless otherwise indicated (* p < 0.05).

In addition to mitochondrial dysfunction, increased ROS levels were also evident in A53TSyn neurons relative to WT. DMF treatment attenuated this increase resulting in ROS levels that were not different from WT controls. DMF treatment did not alter ROS levels in WT neurons. ML385 treatment further increased ROS levels in A53TSyn neurons but had no significant effect in WT neurons (Figure 4).

### 3.4. Modulation of NRF2 Activity Affects Synaptic Plasticity

**Figure 5:**
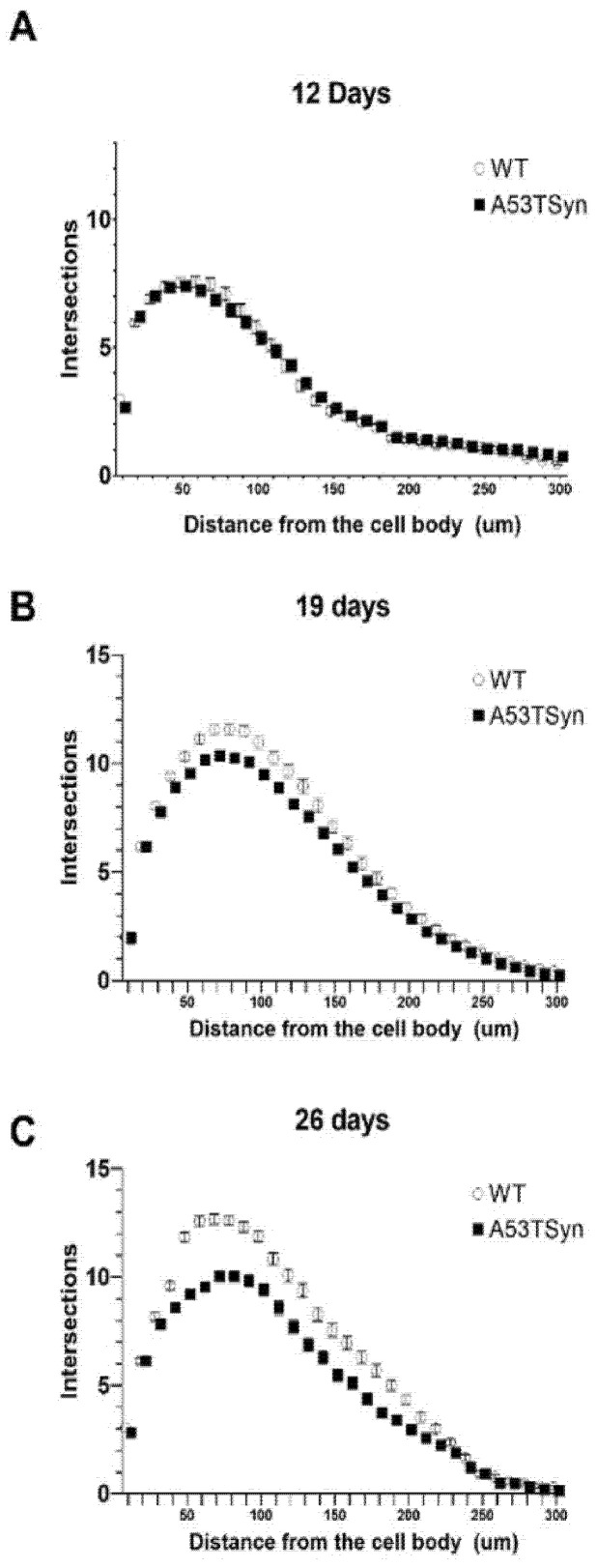
A53TSyn neurons exhibit reduced dendritic complexity with longer culture time. After 12 DIV no genotype differences in arborization were observed between A53TSyn and WT hippocampal neurons (**A**). At 19DIV reduced arborization began to emerge in A53TSyn neurons (**B**) and this reduction is even more pronounced 26DIV (**C**).

Previous studies in our lab and others have shown that hippocampal neurons isolated from mouse models of beta amyloid accumulation exhibit a dystrophic phenotype, characterized by a reduction in dendritic spine density and impaired arborization [31,32,33]. Here, we applied the same techniques to determine if the same dystrophic phenotype could be observed in neurons isolated from a mouse model of synucleinopathy. After 12 days in vitro (DIV) (Figure 5A), no differences in dendritic arborization were seen between A53TSyn and WT neurons, but at 19DIV (Figure 5B), a reduction in arborization was apparent that became even more pronounced after 26DIV (Figure 5C).

**Figure 6:**
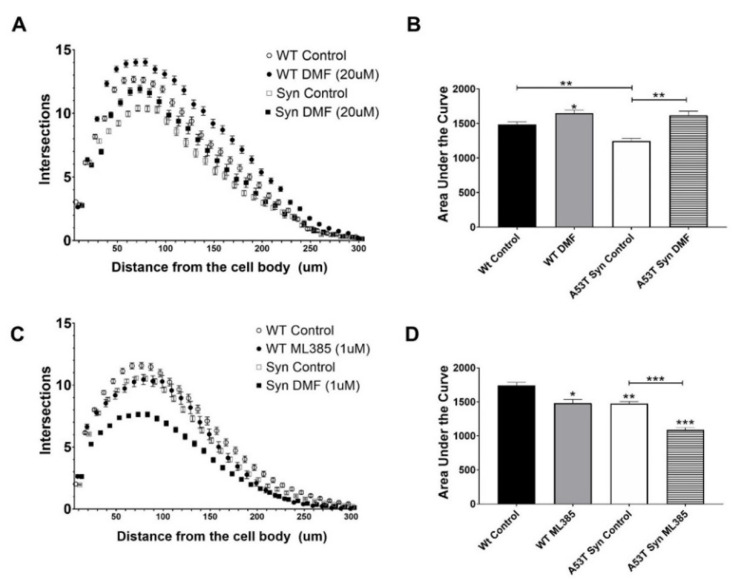
DMF improves dendritic complexity in A53TSyn and WT hippocampal neurons. At 26 DIV, DMF treatment eliminated the deficit in arborization in A53TSyn hippocampal neurons relative to WT neurons (**A**,**B**). Conversely A53TSyn neurons treated with ML385 exhibited an even greater reduction in dendritic arborization at 19DIV than untreated A53TSyn neurons (**C**,**D**). Significance is relative to control unless otherwise indicated (* p < 0.05, ** *p* < 0.01, *** *p* < 0.001).

One week of treatment of neurons with DMF beginning at day 19 attenuated the deficit in arborization in A53TSyn neurons by day 26 back to the same levels as WT. A similar increase in arborization was also seen with DMF treatment in WT neurons (Figure 6A,B). In contrast, arborization at day 19 following ML385 administration on day 12 resulted in exacerbated impairment in arborization in A53TSyn neurons such that on 19 DIV the difference between genotypes were comparable to what was seen between the untreated A53TSyn neurons and WT neurons after 26 DIV (Figure 6C). ML385 treatment likewise impaired arborization in WT neurons as well (Figure 6D) indicating that NRF2 activity can affect dendritic arborization. Because we observed an effect of ML385 in WT neurons without any exposure to an oxidative insult, we wanted to examine if that could be explained by an effect of ML385 treatment on *Keap1* expression. However, ML285 did not significantly alter *Keap1* expression in neurons of either genotype indicating that the negative effect on arborization seen in WT neurons was not the result of changes in *Keap1* levels (Supplementary Figure S1).

**Figure 7:**
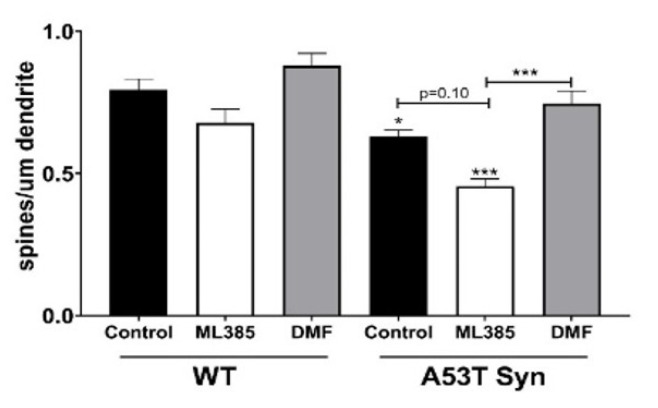
DMF increases spine density in A53TSyn neurons while ML385 treatment reduces it. Significance is relative to control unless otherwise indicated (* p < 0.05, *** *p* < 0.001).

Dendritic spine density was quantified at 14 DIV and found to be significantly decreased in A53TSyn neurons. DMF treatment of A53T neurons restored dendritic spine density to a similar level of the control WT neurons. Although not statistically significant, following ML385 treatment, there was a trend toward an even greater decrease in spine density (Figure 7). Neither ML385 nor DMF treatment had a significant effect on spine density in WT neurons.

## 4. Discussion

In this study we found that modulating NRF2 activity had a significant effect on mitochondrial function, oxidative stress, and synaptic plasticity in aSyn overexpressing neurons. The A53TSyn mouse overexpresses a human aSyn gene with the A53T mutation. These animals develop severe motor impairments at approximately one year of age as well as profound synucleinopathy and cognitive deficits around the same age [34]. We observed that in hippocampal neurons isolated from these animals, there were significant deficits in dendritic arborization and spine density relative to WT neurons. Diminished mitochondrial function and increased intracellular ROS was also seen in the isolated A53TSyn neurons. We found that DMF activated NRF2 in primary hippocampal neurons and this activation attenuated the synaptic deficits observed in A53TSyn neurons, restored mitochondrial function and normalized ROS levels. NRF2 inhibition with the compound ML385 exacerbated the existing deficits in A53TSyn neurons.

To our knowledge, this is the first report of deficits in spine density and arborization in primary A53TSyn neurons in culture. However, similar reductions in spine density were reported in the caudate putamen of 8- and 4-month old A53TSyn mice in vivo [35]. Similarly, in an inducible model of aSyn overexpression, aSyn accumulation was correlated with structural synaptic deficits, and suppression of the aSyn expression reversed these synaptic deficits [36].

Likewise, we believe this is the first report of bioenergetics deficits and increased oxidative stress in isolated A53TSyn neurons. It has been reported that aged A53TSyn mice exhibit mitochondrial abnormalities that are apparent at 11–14 months of age [34]. Our findings are also similar to mitochondrial deficits observed in human neuroblastoma cells overexpressing the A53T or A30P mutations in aSyn, where increased ROS and reduced oxygen consumption were seen relative to cells expressing WT aSyn [37]. Increased oxidative stress has also been observed in blood leukocytes of PD patients [38] and mitochondrial dysfunction, particularly impairments in complex I has been widely reported in the PD brain [39].

The beneficial effects of NRF2 activation on mitochondrial function and oxidative stress that we observed in this study are consistent with previous research on DMF and other NRF2 activating compounds in cellular models of PD. For example, in SH-SY5Y cells, DMF reduced ROS levels and protected against cytotoxicity caused by 6-hydroxydopamine (6-OHDA) treatment [40]. DMF has also been shown to improve mitochondrial function and induce mitochondrial biogenesis in healthy human cells [41]. Another NRF2 activating compound tert-butylhydroquinone (tBHQ) was able to reverse increased ROS and diminished mitochondrial function in in A53T mutant aSyn-expressing N2a cells [42]. The mechanism by which NRF2 activation improves mitochondrial function is not clear. It has been hypothesized that this may have to do with effects on aSyn clearance. In fact, it has recently been shown that NRF2 activation by tBHQ resulted in reduced aSyn levels, however, this effect was observed only in astrocytes and not in neurons [43]. It is unlikely, therefore, that the beneficial effects on NRF2 activation seen in this study is the result of effects on aSyn clearance since our experiments were carried out in neurons grown in isolation. However, this limitation of the present study could be investigated in future work using primary neurons grown in co-culture with astrocytes.

The findings from this study are also in line with previous the reported in vivo effects of DMF in models of PD and other neurodegenerative disease. In the MPTP (1-methyl-4-phenyl-1,2,3,6-tetrahydropyridine) neurotoxin-induced model of PD, NRF2 activation by DMF similarly reduced oxidative damage and decreased aSyn accumulation [44]. Similarly, oral treatment of DMF in 6-OHDA-treated mice induced expression of NRF2-regulated genes, and protein levels of NRF2 and attenuate 6-OHDA induced oxidative stress and neuroinflammation [40]. DMF treatment also seems to be beneficial for PD-related motor deficits. In a PD model using AAV mediated aSyn overexpression, DMF treatment decreased motor deficits and reduced dopamine cell loss [20].

Our findings that NRF2 inhibition exacerbated impairments in mitochondrial function, oxidative and synaptic density in A53TSyn neurons supports the existing literature describing the effects of NRF2 inhibition by ML385 in other contexts. Our lab has previously shown that inhibition of NRF2 with ML385 resulted in heightened levels of intracellular ROS in cortical neurons isolated from the 5xFAD mouse model of beta amyloid accumulation [45]. In vivo, ML385 administration was also found to increase oxidative stress and inflammation in otherwise healthy mice [46]. The inhibition of NRF2 by ML385 occurs via direct binding to the protein which interferes with its ability to bind to target DNA sequences [47]. Our finding that ML385 does not affect *Keap1* expression is in line with this reported mechanism of NRF2 inhibition. The fact that we observed impaired dendritic arborization in WT neurons not exposed to an exogenous stressor suggests a role for NRF2 in regulating synaptic plasticity under normal conditions.

Such a role for NRF2 is also supported by previously published data from our own lab showing that hippocampal neurons isolated from NRF2 knockout (NRF2KO) mice display reduced dendritic complexity and synaptic density relative to WT neurons [22]. Increased ROS and deficits in mitochondrial function was also observed in NRF2KO neurons compared to WT neurons [22]. Loss of NRF2 in the context of PD has also led to deleterious effects on oxidative stress and neuronal health. A recent study found that human aSyn overexpressed in NRF2KO mice induced even worse oxidative damage than seen in the aSyn overexpressing mice that did express NRF2 [19]. Similarly, loss of NRF2 was shown to result in exacerbated loss of dopamine neurons and more pronounced motor deficits in an AAV-mediated aSyn overexpression model [40].

The effects we observed in this study of modulating NRF2 activity on synaptic plasticity suggest that the same modulation could have significant consequences for cognitive function in PD as synaptic density correlates very strongly with cognitive function in both rodents and humans [22,24,48,49,50]. Although, to our knowledge, the effects of modulating NRF2 activity on cognition have not been specifically investigated in PD models, there is evidence in aging and other neurodegenerative disease that NRF2 plays a role in maintaining cognitive function. Our own lab has demonstrated that loss of NRF2 results in accelerated cognitive decline during aging in mice [21]. Conversely, activation of NRF2 results in cognitive enhancement in a variety of conditions associated with cognitive impairment. Treatment with a NRF2 activating extract of the plant *Centella asiatica* has been shown to improve learning, memory, and executive function in mouse models of aging and beta amyloid accumulation [25,31]. Sulforaphane, another potent NRF2 activating compound found in cruciferous vegetables, ameliorated cognitive deficits in mouse models of Alzheimer’s disease, traumatic brain injury and vascular cognitive impairment [51,52,53,54]. Cognitive enhancing effects of DMF have also been described in rodent models of sepsis and vascular dementia [55,56]. Even more convincing perhaps is a recent finding from a clinical trial of DMF in multiple sclerosis patients showing slowed cognitive decline over two years of treatment [57]. This strongly suggests that NRF2 activation could be a viable therapeutic strategy for cognitive enhancement in a broad range of diseases.

## 5. Conclusions

In summary, we have shown that activation of NRF2 can improve mitochondrial and synaptic impairments in A53Tsyn hippocampal neurons, while inhibition of NRF2 exacerbates these endpoints. Future studies are needed to confirm these effects in vivo in this and other models of PD to determine the therapeutic utility of targeting NRF2 to improve cognitive function in PD.

## Data Availability

Data is contained within the article.

## Supplementary Materials

The following supporting information can be downloaded at: https://www.mdpi.com/article/10.3390/antiox11010026/s1, Figure S1: *Keap1* gene expression in primary neurons.
