# Supplementary material for: NRF2 Activation Ameliorates Oxidative Stress and Improves Mitochondrial Function and Synaptic Plasticity, and in A53T α-Synuclein Hippocampal Neurons
Source: Antioxidants (Basel). 2021 Dec 24;11(1):26. doi: 10.3390/antiox11010026 (PMC8772776; doi:10.3390/antiox11010026)
Supplement: Supplementary file 1 [file antioxidants-11-00026-s001.zip › Supplementary File.pdf]

Supplementary File

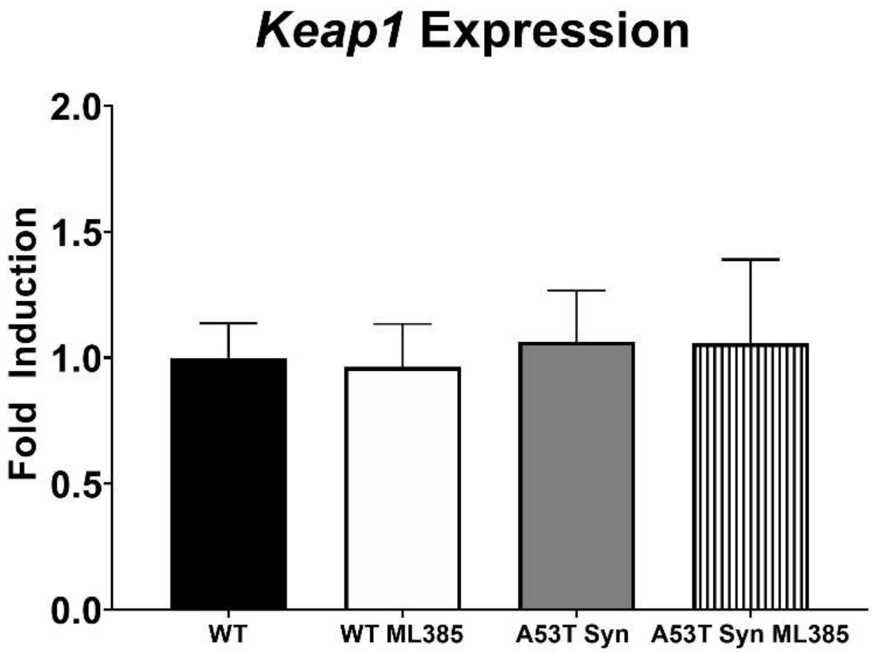

Supplementary Figure S1: *Keap1* gene expression in primary neurons. *Keap1* gene expression in A53TSyn neurons was no different from expression in WT neurons. Treatment with ML385 did not effect expression in either genotype (*n* = 6–10).
